# Cre-dependent ACR2-expressing reporter mouse strain for efficient long-lasting inhibition of neuronal activity

**DOI:** 10.1038/s41598-023-30907-2

**Published:** 2023-03-09

**Authors:** Yasutaka Mukai, Yan Li, Akiyo Nakamura, Noriaki Fukatsu, Daisuke Iijima, Manabu Abe, Kenji Sakimura, Keiichi Itoi, Akihiro Yamanaka

**Affiliations:** 1 Department of Neuroscience II, Research Institute of Environmental Medicine, Nagoya University, Nagoya, 464-8601 Japan; 2 Department of Neural Regulation, Nagoya University Graduate School of Medicine, Nagoya, 466-8550 Japan; 3 Department of Animal Model Development, Brain Research Institute, Niigata University, Niigata, 951-8585 Japan; 4 Department of Nursing, Faculty of Health Sciences, Tohoku Fukushi University, Sendai, 981-8522 Japan; 5 Chinese Institute for Brain Research, Beijing, 102206 China; 6 National Institute for Physiological Sciences, National Institutes of Natural Sciences, Aichi, 444-8585 Japan; 7 Department of Neuropsychiatry, Keio University School of Medicine, Tokyo, 160-8582 Japan

**Keywords:** Circadian rhythms and sleep, Neural circuits, Neuronal physiology

## Abstract

Optogenetics is a powerful tool for manipulating neuronal activity by light illumination with high temporal and spatial resolution. Anion-channelrhodopsins (ACRs) are light-gated anion channels that allow researchers to efficiently inhibit neuronal activity. A blue light-sensitive ACR2 has recently been used in several in vivo studies; however, the reporter mouse strain expressing ACR2 has not yet been reported. Here, we generated a new reporter mouse strain, *LSL-ACR2*, in which ACR2 is expressed under the control of Cre recombinase. We crossed this strain with a noradrenergic neuron-specific driver mouse (*NAT-Cre*) to generate *NAT-ACR2* mice. We confirmed Cre-dependent expression and function of ACR2 in the targeted neurons by immunohistochemistry and electrophysiological recordings in vitro, and confirmed physiological function using an in vivo behavioral experiment. Our results show that the *LSL-ACR2* mouse strain can be applied for optogenetic inhibition of targeted neurons, particularly for long-lasting continuous inhibition, upon crossing with Cre-driver mouse strains. The *LSL-ACR2* strain can be used to prepare transgenic mice with homogenous expression of ACR2 in targeted neurons with a high penetration ratio, good reproducibility, and no tissue invasion.

## Introduction

Manipulation of neuronal activity is indispensable for understanding the causal relationships between neuronal activity and outcomes. Optogenetics is a powerful tool for manipulating neuronal activity by light illumination with high temporal and spatial resolution. Opsins are a group of membrane proteins that are activated by light. Channelrhodopsin-2 (ChR2), which is the most well known opsin, is a blue light-gated cation channel that allows for cation inflow to induce depolarization and excitation of neurons^1^. ChR2-mediated neuronal excitation can often indicate the “sufficiency” of specific neuronal activity for a specific outcome^2^. On the other hand, the “necessity” of specific neuronal activity is often demonstrated by neuronal inhibition. Archaerhodopsin (Arch) and halorhodopsin (Halo) are commonly used light-driven proton and chloride pumps, respectively, that can induce hyperpolarization and inhibition of neurons^3–5^. These pump-mediated neuronal inhibitions can demonstrate the “necessity” of specific neuronal activity for a specific outcome^6–9^. However, as both Arch and Halo are pumps, and not light-driven channels, photocurrents elicited by either of them are lower than those of ion channels.

The discovery of *Guillardia theta* anion-channelrhodopsins (ACRs)^10^ and the development of chloride-conducting channelrhodopsins^11^ enabled researchers to overcome the above-mentioned difficulties in optogenetic neuronal inhibition. ACRs are light-gated anion channels that can induce anion transduction, leading to hyperpolarization and inhibition of neurons with a low intracellular chloride ion (Cl^−^) concentration ([Cl^−^]). There are two main subtypes of ACRs, namely ACR1 and ACR2, with the major difference between the subtypes being the maximally sensitive light wavelength, which is 515 nm for ACR1 and 470 nm for ACR2^10^.

Compared with the transgene introduction by viral vector injection^12^, transgenic mouse lines have the advantage of homogeneous gene expression in targeted neurons with a higher penetration ratio, better reproducibility, and no tissue invasion. A reporter mouse strain expressing ACR1 under the control of Cre recombinase was reported recently^13^, but a reporter mouse strain expressing ACR2 has not been reported yet. In the present study, we generated a new mouse strain in which ACR2 is expressed under the control of Cre recombinase, and confirmed that a selective neuronal population is inhibited by light exposure both in vitro (using slice patch clamp recording) and in vivo (via a behavioral experiment). Since ACR2 is activated by blue light, the present mouse strain can be utilized with the same experimental setup for ChR2.

## Results

### *NAT-ACR2* mice expressing ACR2 in LC-NA neurons

**Figure 1:**
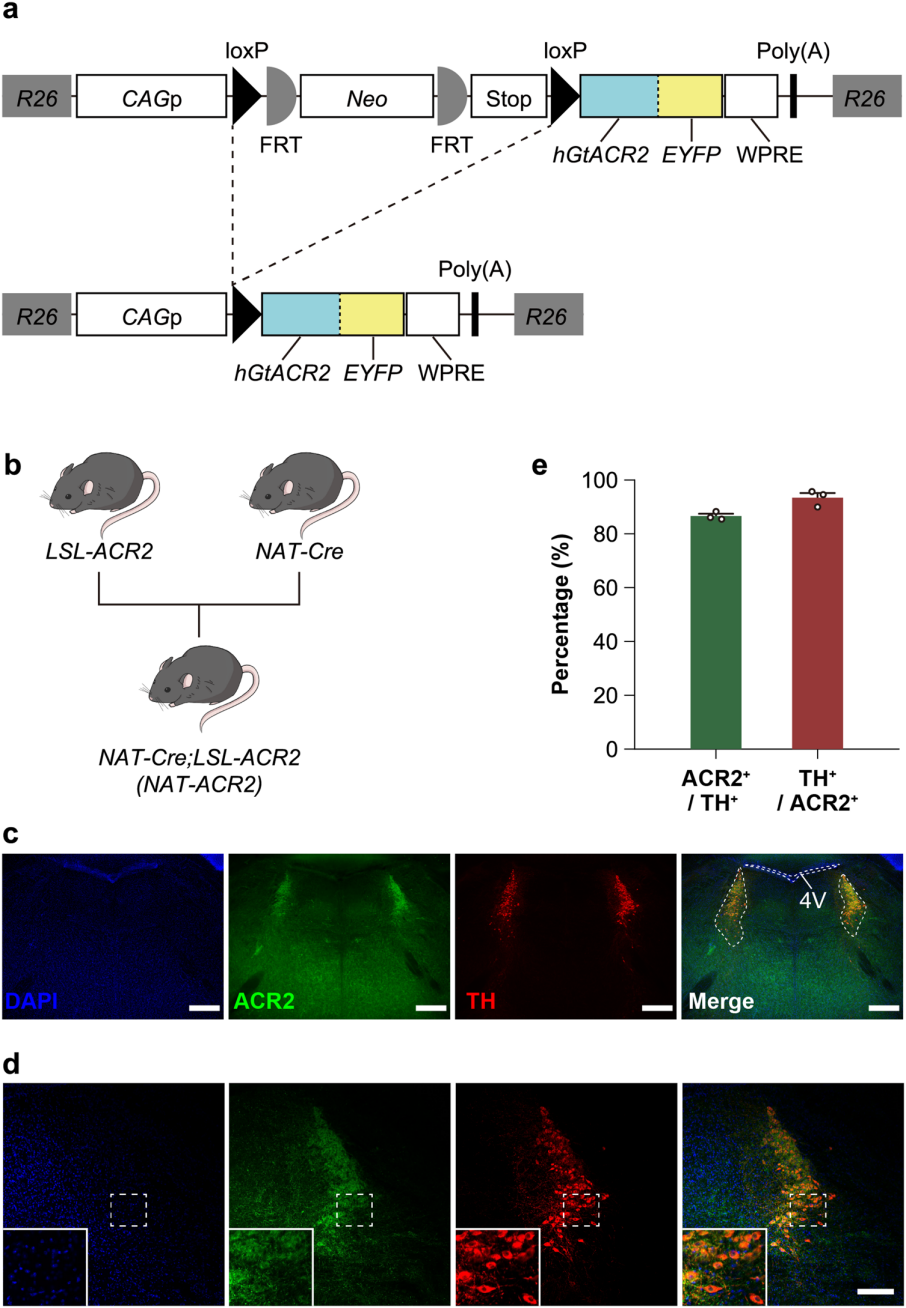
The *LSL-ACR2* mouse strain. (**a**) Schematic showing the transgene construction of *LSL-ACR2* transgenic mice at *Rosa26* locus. *R26,* Rosa26 locus; *CAGp*, CAG promoter; *loxP,* loxP sequence; *FRT,* flippase recognition target sequence; *Neo,* neomycin resistant gene; *Stop,* stop cassette; *hGtACR2,* human codon-adapted anion channelrhodopsin 2 gene; *EYFP,* enhanced yellow fluorescent protein; *WPRE,* woodchuck hepatitis virus posttranscriptional regulatory element; *Poly(A),* poly-adenylation sequence. (**b**) *LSL-ACR2* and *NAT-Cre* mice were crossed to generate *NAT-Cre*; *LSL-ACR2* (*NAT-ACR2*) mice. (**c**) Representative immunostaining image of *NAT-ACR2* neurons visualized in the LC. Blue: DAPI, Green: ACR2, Red: TH, scale bar: 500 μm. (**d**) Representative immunostaining image of *NAT-ACR2* neurons in the right side of the brain visualized in the LC. Insets are magnified images of the dotted area. Blue: DAPI, Green: ACR2, Red: TH, scale bar: 200 μm. (**e**) Proportion of positive cells expressing ACR2 and TH in *NAT-ACR2* mice (n = 3 animals). ACR2^+^/TH^+^: ACR2^+^ in TH^+^ cells, 86.6 ± 0.8%. TH^+^/ACR2^+^: TH^+^ in ACR2^+^ cells, 93.4 ± 1.8%.

To express ACR2 exclusively in Cre recombinase-expressing cells, we generated a new mouse strain, namely *Rosa26-CAGp-LSL-ACR2-EYFP* (*LSL-ACR2*, Fig. 1a; see also “Materials and methods”). To examine the expression and function of ACR2, we crossed *LSL-ACR2* with the *noradrenaline-transporter (NAT)-Cre* (*NAT-Cre*) mouse strain^14^, which expresses Cre recombinase in noradrenergic (NA) neurons, to produce the *NAT-Cre;LSL-ACR2* (*NAT-ACR2*) mouse strain (Fig. 1b). We examined the expression of ACR2 by immunohistochemistry. ACR2-positive (ACR2^+^) cells were visualized by fused enhanced yellow fluorescent protein (EYFP) and observed in the locus coeruleus (LC), which is a major nucleus of NA neurons (LC-NA neurons) in the forebrain (Fig. 1c,d). ACR2^+^ cells overlapped with tyrosine-hydroxylase positive (TH^+^) cells at a high rate, indicating that they are mostly NA neurons in the LC (coverage: ACR2^+^ in TH^+^, 86.6 ± 0.8%; specificity: TH^+^ in ACR2^+^, 93.4 ± 1.8%; TH^+^, 273.3 ± 19.6 cells; ACR2^+^, 253.0 ± 14.2 cells; TH^+^ and ACR2^+^, 236.7 ± 17.4 cells; n = 3 animals; Fig. 1e). We also observed a characteristic pattern of ACR2 expression in siblings of *LSL-ACR2* mice crossed with *Vglut2-Cre*, *Vgat-Cre*, and *DAT-Cre* mice, which express Cre recombinase in glutamatergic, GABAergic, and dopaminergic neurons, respectively (Supplementary Fig. S1). On the other hand, we did not observe any ACR2-EYFP-derived fluorescence in the brains of Cre-negative *LSL-ACR2* mice (n = 4 animals; Supplementary Fig. S2). Taken together, these results suggest that the *LSL-ACR2* mouse strain efficiently and exclusively expresses ACR2 in a Cre-dependent manner.

### Light-induced transient and sustained photocurrents

Next, we examined the function of ACR2 in LC-NA neurons by patch-clamp recording in acute brain slices. Irradiation of ACR2 at a wavelength of 470 nm was previously found to result in rapid inflow of Cl^−^ and inhibition of neuronal activity^10^. Therefore, we performed patch clamp recording with the whole-cell recording mode using a pipette solution with a low Cl^−^ concentration (8 mM). A brain slice was anchored in a chamber perfused with artificial cerebrospinal fluid (aCSF). Under an epifluorescent microscope, to minimize photoactivation of ACR2, very brief low intensity 505 nm LED illumination (74 µW/mm^2^, 100 ms) was used to visualize native EYFP fluorescence from ACR2-EYFP as an LC-shaped faint signal, since ACR2-EYFP was expressed on the cellular membrane. We identified LC-NA neurons by a combination of anatomical location, triangular cellular shape, and fluorescence observed in the recording area. In the following experiments, 470 nm light at multiple light intensities (ranging from 1 to 100%) were used to activate ACR2 at 0.011 to 3.1 mW/mm^2^.

**Figure 2:**
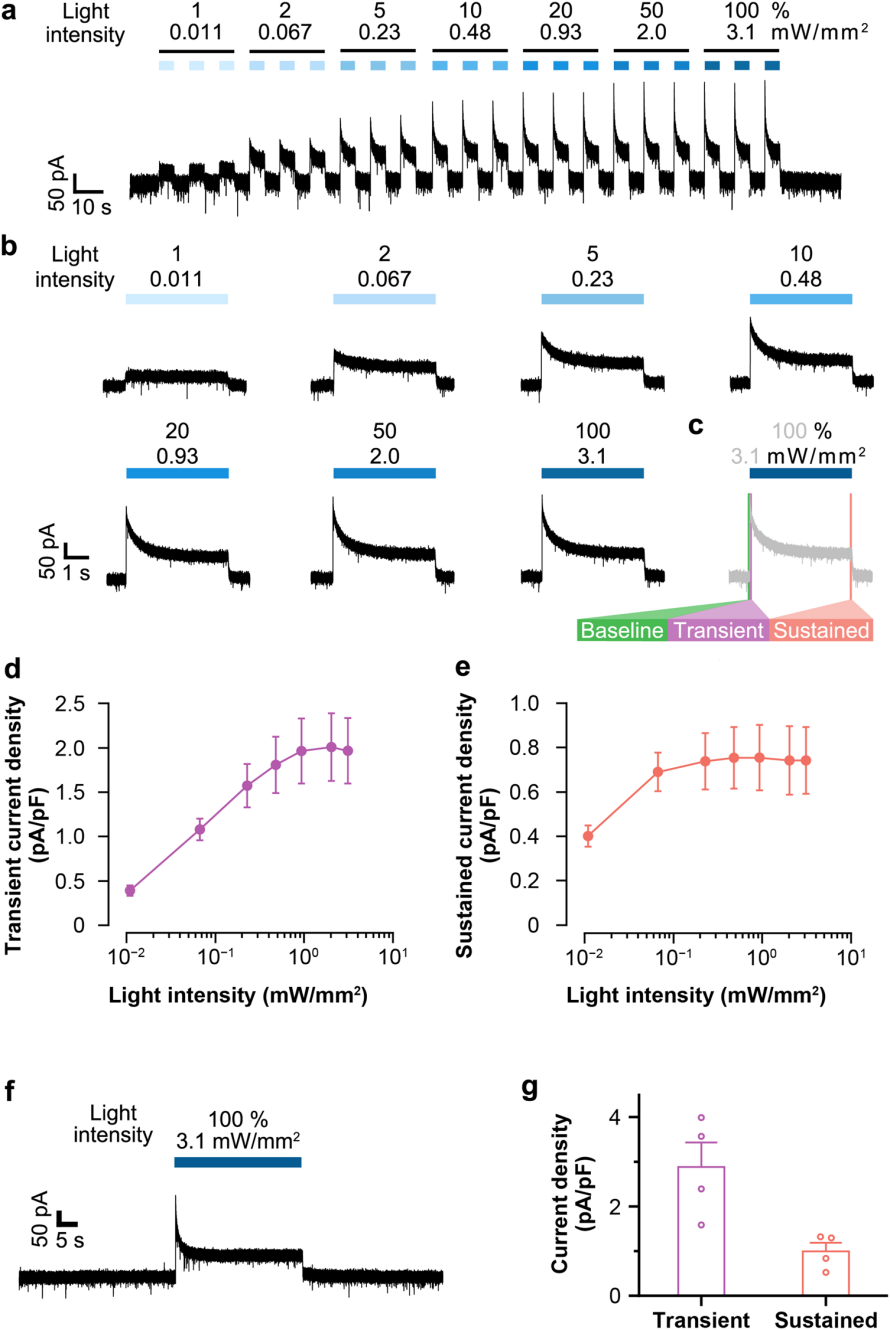
Light-induced photocurrents in ACR2-expressing neurons. (**a**) Representative trace of photocurrent recording with illumination at different intensities of 470 nm light. Membrane potential was held at − 60 mV. Light intensity was adjusted from 1 to 100%, corresponding to 0.011 to 3.1 mW/mm^2^, as indicated above the trace. (**b**) Single trace of photocurrent recording during illumination at different light intensities. (**c**) Definition of baseline, transient, and sustained currents. Baseline: 100 ms before light onset for each illumination. Transient: 100 ms after light onset for each illumination. Sustained: 100 ms before light offset for each illumination. (**d**,**e**) Transient and sustained current densities as a function of light intensity in (**d**) and (**e**), respectively. Dots show mean values ± SEM of 9 cells from 2 animals. (**f**) Representative trace of photocurrent recording at 470 nm, 30 s light illumination; the membrane potential was held at − 60 mV. Light intensity: 100%, corresponding to 3.1 mW/mm^2^. (**g**) Transient and sustained current densities (n = 4 cells from an animal). Dots show individual data.

Illumination with different intensities of 470 nm light (3 consecutive light exposures with 5-s intervals) induced outward photocurrents, which were recorded by holding the membrane potential at − 60 mV (Fig. 2a). The effect of light intensity on the photocurrent amplitude is shown in the single trace recording in Fig. 2b**.** To further confirm the correlation between amplitude and light intensity, we defined the baseline, transient current, and sustained current as noted in Fig. 2c. Given that cell size affects the amount of ACR2 expression, we examined current density (i.e., photocurrent amplitude divided by membrane capacity), because absolute value of the current depends on cell size. Indeed, the current density was found to be positively correlated with the light intensity (Fig. 2d,e). Consistent with the results of a previous study^10^, the transient photocurrent reached the saturation level at 20% (0.93 mW/mm^2^) light intensity (Fig. 2d), and the sustained photocurrent reached the saturation level at 5% (0.23 mW/mm^2^) light intensity (Fig. 2e). Furthermore, transient and sustained photocurrents were also induced by long-term (30 s) light illumination (Fig. 2f,g). These results demonstrate that ACR2 in LC-NA neurons was functional, and that it induced an outward current upon illumination with 470 nm light.

### ACR2 inhibits neuronal activity by light-induced chloride ion flow

**Figure 3:**
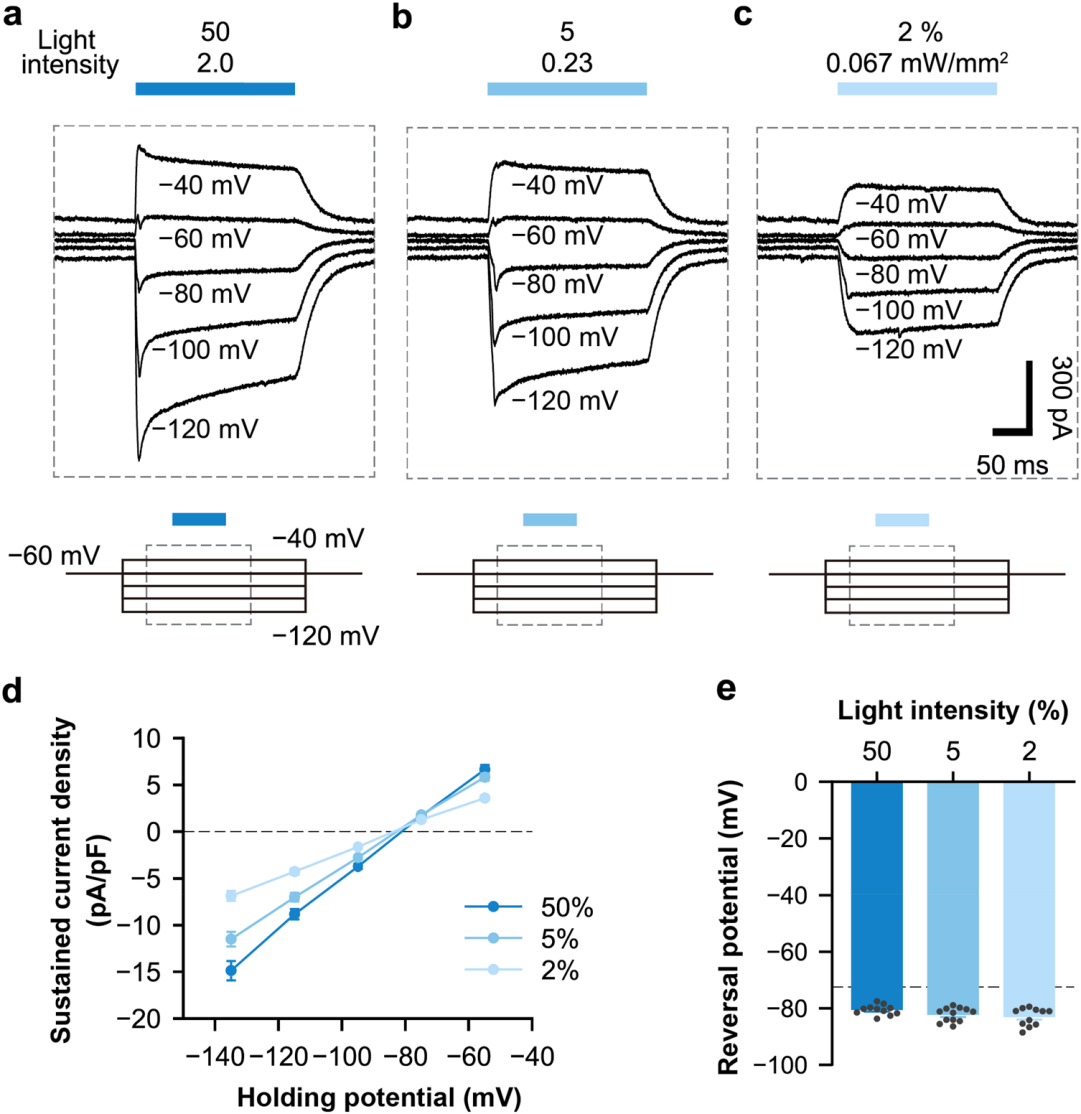
ACR2-induced chloride ion inflow and outflow upon illumination. (**a**–**c**) Upper: representative traces of photocurrent recording with illumination at different intensities of 470 nm light held at − 120 mV to − 40 mV. Bottom: schematic of the command voltage. The dotted rectangle indicates the timing of the upper traces. Light intensity: 50%, 2.0 mW/mm^2^ (**a**); 5%, 0.23 mW/mm^2^ (**b**); 2%, 0.067 mW/mm^2^ (**c**). (**d**) Sustained current densities as a function of the holding potential. The holding potential was corrected with the junction potential (− 14.9 mV). The dots show mean values ± SEM. Light intensity at 50%, n = 11 cells; 5%, n = 12 cells; 2%, n = 11 cells from 2 animals. The dotted line indicates 0 pA/pF. (**e**) The reversal potential for different light intensities is based on each recording by linear fitting. Bars show the mean values ± SEM. Dots show individual values from each cell. The dotted line indicates the reversal potential of Cl^−^ (− 72.6 mV).

To further confirm whether ACR2 generates outward photocurrent by inducing the inward flow of Cl^−^, we investigated the reversal potential of the photocurrent ions by voltage clamp step recording. Light illumination (200 ms, 470 nm) at 50% (2.0 mW/mm^2^), 5%, and 2% (0.067 mW/mm^2^) intensity induced different degrees of outward or inward photocurrent under a voltage step from − 120 to − 40 mV (Fig. 3a–c). We further analyzed the correlation between sustained photocurrent and holding potential (Fig. 3d) and the reversal potential was calculated by fitting a straight line (Fig. 3e). The holding potential was adjusted with the calculated junction potential (− 14.9 mV). Higher light intensity produced higher sustained induced current density, which was consistent with our previous results (Fig. 2d,e). All reversal potentials of the photocurrent ions during 50% (− 80.7 ± 0.5 mV, n = 11 cells), 5% (− 82.4 ± 0.7 mV, n = 12 cells), and 2% (− 83.1 ± 1.0 mV, n = 11 cells) light illumination were similar to the theoretical reversal potential of Cl^−^ (− 72.6 mV) (Fig. 3e) under the recording conditions.

**Figure 4:**
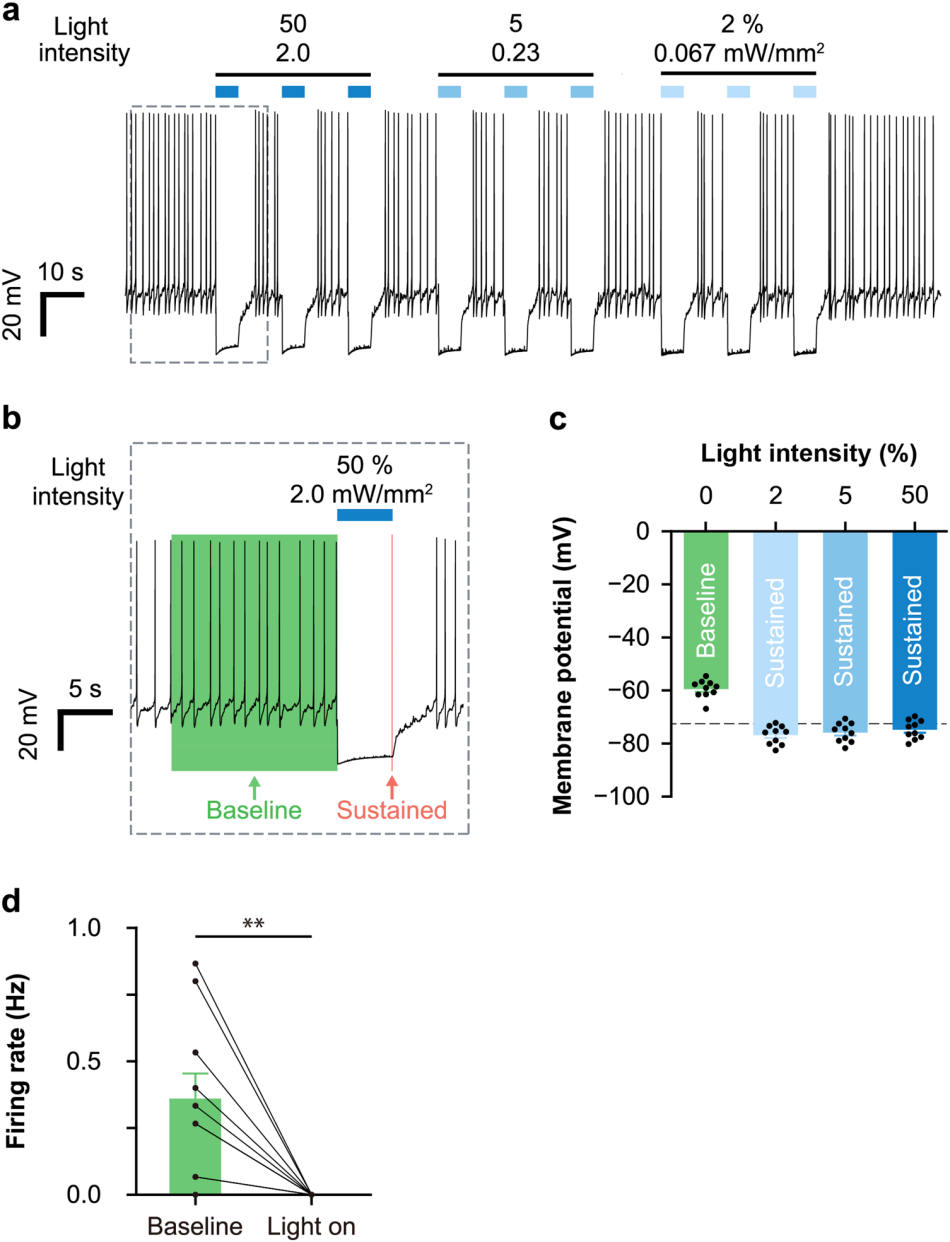
ACR2-inhibited neuronal activity by chloride ion inflow upon illumination. (**a**) Representative trace of membrane potential recording with illumination at different intensities of 470 nm light. Light intensity was adjusted from 50 to 2%, corresponding to 2.0 to 0.067 mW/mm^2^, as indicated above the trace. Data within the dotted rectangle is also shown in (**b**). (**b**) Definition of baseline and sustained membrane potential. Baseline: 15 s before light onset for the first illumination of the session. Sustained: 100 ms before light offset for each illumination. (**c**) The reversal potential for different light intensities was calculated based on current clamp recording (n = 10 cells from 2 animals). Bars show mean values ± SEM. Dots show individual data from each cell. Dotted line: Reversal potential of Cl^−^ (− 72.6 mV). (**d**) Comparison of neuronal firing rate before and after light onset (n = 10 cells from 2 animals). **p < 0.01, paired t-test. The baseline was defined as shown in (**b**). Bars show mean values ± SEM. Dots show individual cell data including all light intensities.

We also examined membrane potential change by current clamp recording during light illumination (200 ms, 470 nm) at 50%, 5%, and 2% intensity (Fig. 4a). To analyze the amplitude of the membrane potential change, we defined the baseline potential as the median potential during 15 s before light onset of the first illumination of the session (− 59.5 ± 1.1 mV, n = 10 cells), and the sustained potential as the mean of 100 ms before light offset of each illumination (Fig. 4b). Similar to the voltage clamp step recordings, the reversal potentials of the photocurrent ions at 50% (− 74.8 ± 1.1 mV, n = 10 cells), 5% (− 75.9 ± 1.1 mV, n = 10 cells), and 2% light illumination (− 76.8 ± 1.1 mV, n = 10 cells) were close to the theoretical reversal potential of Cl^−^ (− 72.6 mV) (Fig. 4c) under the recording conditions. These results suggest that ACR2 inhibited cellular activity by inducing chloride ion inflow upon light illumination in LC-NA neurons.

### ACR2 induced durable and long-lasting inhibition of neuronal activity

**Figure 5:**
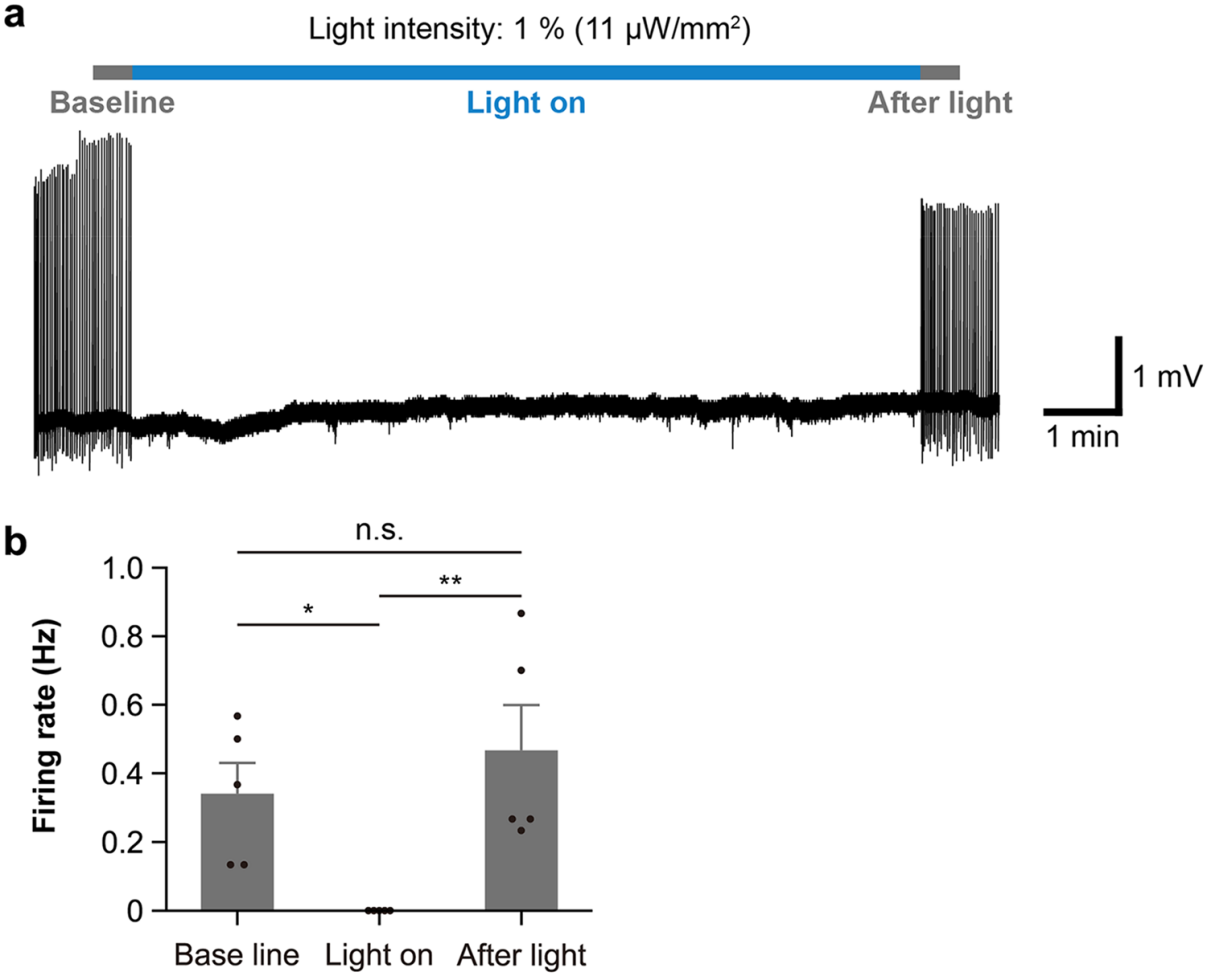
ACR2 continuously inhibited neuronal activity. (**a**) Representative trace of loose cell recording at 470 nm, 10 min light illumination. Light intensity was 1%, corresponding to 11 µW/mm^2^. (**b**) Comparison of the neuronal firing rate before, during, and after light illumination (n = 5 cells from an animal). 'Baseline' and 'after light' were defined as 30 s before and after light illumination, respectively. *p < 0.05, **p < 0.01, n.s., not significant, Tukey’s test. The bars show mean values ± SEM. The dots show individual cell data.

To further confirm the influence of ACR2 on neuronal activity, we also examined the change in neuronal firing by current clamp recording. We found that light illumination with 50%, 5%, and 2% intensity caused hyperpolarization and completely inhibited generation of action potential (paired t-test, p = 0.004, n = 10 cells, Fig. 4d). In the loose cell-attached mode recording, which does not rupture the membrane and does not alter the natural intracellular environment, ACR2 also inhibited neuronal firing during long-term light illumination (11 µW/mm^2^, 10 min) and did not significantly affect the firing rate after illumination (Tukey’s test; baseline vs. light on, p = 0.02; light on vs. after light, p = 0.004; baseline vs. after light, p = 0.45; n = 5 cells, Fig. 5a,b). These results show that ACR2 could inhibit cellular activity in LC-NA neurons, and did not affect neuronal activity after optogenetic inhibition for a long period of time.

### ACR2 was functional in free-moving animals in vivo

**Figure 6:**
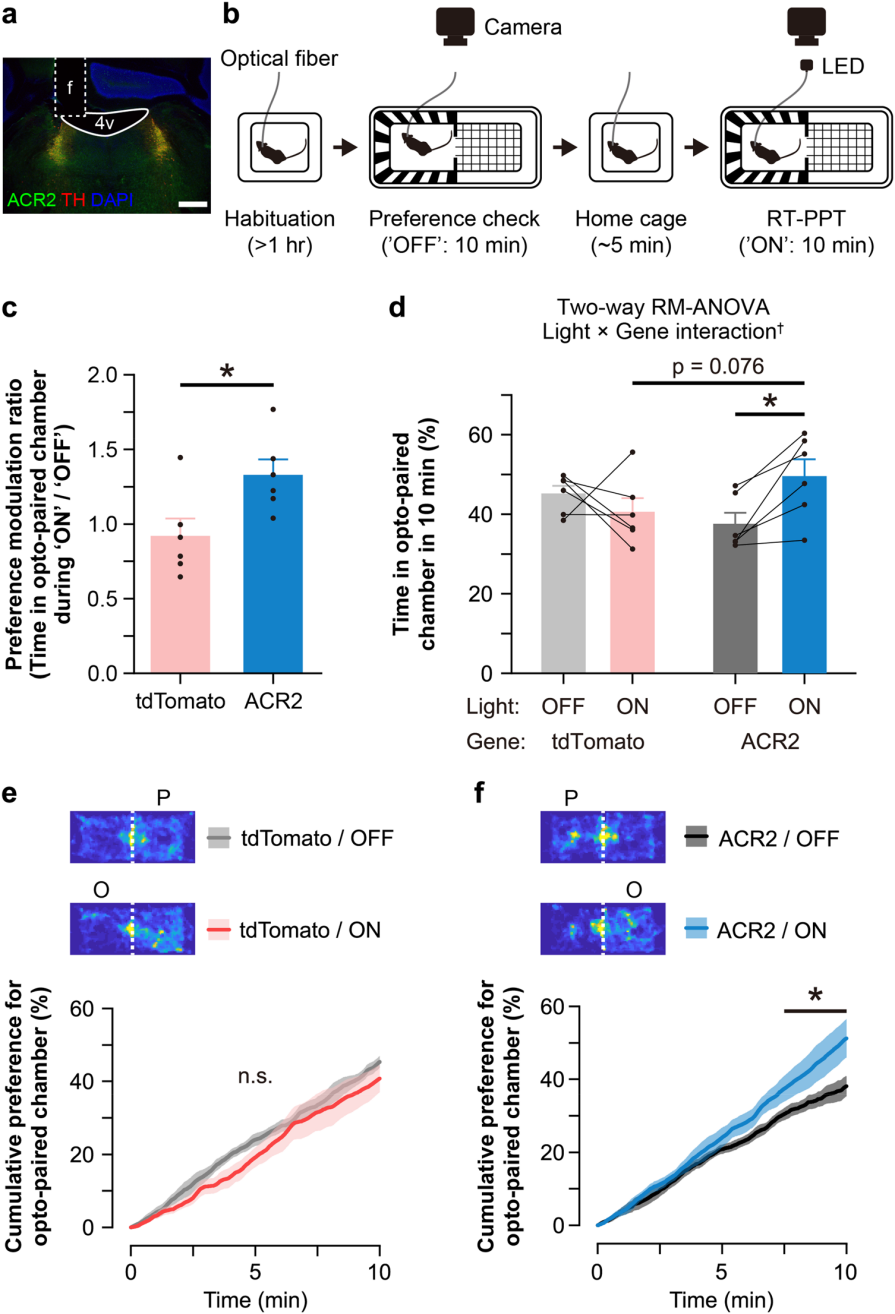
In vivo inhibition of LC-NA neuron-induced place preference. (**a**) Representative brain slice image showing the position of the implanted fiber above the LC. f, fiber tract; 4v, fourth ventricle. Green: ACR2, Red: TH, Blue: DAPI, scale bar: 500 μm. (**b**) Schematic of real-time place preference test (RT-PPT). After an animal was habituated with an optic fiber for > 1 h in the home cage (Habituation), the place preference at baseline was examined for 10 min in the two-chamber apparatus (Preference check, ‘OFF’). Then, the animal was temporarily placed back into the home cage and preference analysis was performed after ~ 5 min (Home cage). The opto-paired chamber was determined to be the non-preferred chamber, and RT-PPT was performed for 10 min (RT-PPT, ‘ON’). (**c**) Preference modulation ratio of control animals expressing tdTomato and animals expressing ACR2. *p < 0.05 (tdTomato, n = 6 animals; ACR2, n = 6 animals, Welch’s t-test). (**d**) Time spent in the opto-paired chamber during 10 min, or the preference, at baseline (OFF) and RT-PPT (ON). ^†^p < 0.05, significant interaction shown in two-way repeated measures (RM) ANOVA between factors ‘light’ and ‘gene’. *p < 0.05, ‘p = 0.076’, post hoc Tukey’s test. tdTomato, n = 6 animals; ACR2, n = 6 animals. (**e** and **f**) The top heat maps show the time spent in the two-chamber cage for a representative animal from each group. P, preferred chamber; O, opto-paired chamber. The line graph shows the cumulative place preference over 10 min. n.s., not significant (n = 6 animals, Tukey’s test for every 10 s, OFF vs. ON); *p < 0.05 (n = 6 animals, Tukey’s test for every 10 s, OFF vs. ON). Line, mean; shadow, standard error of the mean.

Finally, to confirm the function of ACR2 in vivo, we performed a real-time place preference test (RT-PPT). We implanted an optical fiber cannula above the LC unilaterally in *NAT-ACR2* mice and *NAT-Cre; Ai14* mice (*NAT-tdTomato* mice) (Fig. 6a). *NAT-tdTomato* mice express the red fluorescent protein tdTomato in NA neurons as a negative control. We allowed the animals to explore a two-chamber apparatus for 10 min without light illumination (‘OFF’ session), and identified a preferred chamber at baseline. Then, we allowed the animals to explore the apparatus for 10 min (‘ON’ session), with illumination of continuous light (470 nm) while the animal was in the un-preferred chamber, i.e., the opposite side of the preferred chamber (opto-paired chamber; Fig. 6b). Then, we examined the shift of the preference induced by light illumination in *NAT-ACR2* and *NAT-tdTomato* mice. We calculated the ‘preference modulation ratio,’ which is the time spent in the opto-paired chamber during the ‘ON’ session divided by that during the ‘OFF’ session. We found that the preference modulation ratio was significantly higher in *NAT-ACR2* mice than in *NAT-tdTomato* mice (p = 0.026, Welch’s t-test; Fig. 6c), suggesting that light illumination of ACR2 in *NAT-ACR2* mice significantly increased the animals’ place preference. We also analyzed raw values of the time spent in the opto-paired chamber during ‘OFF’ and ‘ON’ sessions (within-subjects factor ‘light’) of *NAT-ACR2* and *NAT-tdTomato* mice (between-subjects factor ‘gene’) by two-way repeated measures ANOVA. We found a significant interaction between ‘light’ and ‘gene’ factors (p = 0.021). Furthermore, we found a significant difference in the ‘light’ factor in *NAT-ACR2* (p = 0.019, post hoc Tukey’s test), but no significant difference in the ‘gene’ factor during the ‘ON’ session (p = 0.076, post hoc Tukey’s test; Fig. 6d). We suggest that the lack of significance could be due to the increased variance of the place preference among animals during the ‘ON’ session. Finally, we examined the preference change throughout a session. We found that the time spent in the opto-paired chamber during the 10 min experiment of *NAT-tdTomato* mice was unchanged between the ‘OFF’ and ‘ON’ sessions (Fig. 6e), while that of *NAT-ACR2* mice was gradually increased during the ‘ON’ session (Fig. 6f). These results suggest that light illumination of ACR2 expressed in *NAT-ACR2* successfully modulated animals’ behavior. It was also shown that a unilateral inhibition of LC-NA neuronal activity was sufficient to induce place preference.

## Discussion

In this study, we generated and characterized a new mouse strain, namely *LSL-ACR2*, for exclusive inhibition of neuronal activity in Cre-expressing targeting neurons (Fig. 1a). The *NAT-ACR2* mice were generated by crossing *LSL-ACR2* mice with *NAT-Cre* mice. The function of ACR2 in vitro was confirmed by patch-clamp recording, and it was shown that ACR2 activated by 470 nm light could effectively inhibit neuronal activity by inducing Cl^−^ inflow. This study provides the theoretical basis for the use of the *LSL-ACR2* mouse strain in behavioral experiments. To date, ACR2 expression in rodent neurons has been achieved by viral infection^15–19^. To use viral vectors, researchers need to optimize various indefinite factors of the virus, such as the position, volume, titer, and serotype. When using custom-made viral vectors, researchers also need to consider the promoter and other DNA constructs. On the other hand, using the *LSL-ACR2* with Cre-driver mice, researchers can skip all of the above-mentioned processes needed for viral vectors. Thus, the *LSL-ACR2* mouse strain will allow researchers to more conveniently express ACR2 in a specific subtype of neurons with homogenous quantity and quality.

We found that neuronal activity was inhibited via ACR2 by 470 nm light at intensities as low as 11 µW/mm^2^, and that the effects lasted for > 10 min. Remarkably, neuronal activity recovered immediately after the termination of long-term inhibition (Fig. 5). An advantage of ACR2 is the efficiency of phototransduction compared with other inhibitory optogenetic tools, including light-driven pumps such as Arch and Halo, and also gene-engineered chloride-conducting channelrhodopsins, such as slowChloC and iC++^10, 18^. The characteristic lower intensity and longer duration light-inducibility is advantageous for in vivo applications, since it will cause less heat generation and less phototoxicity^20, 21^. Furthermore, lower intensity light inducibility might presumably be applicable for optogenetic manipulation without intracranial surgery like ChRmine and OPN4dC^22, 23^. To inhibit neuronal activity for a prolonged period of time, researchers can also use chemogenetic approaches, such as designer receptors exclusively activated by designer drugs (DREADDs)^24^. Compared to chemogenetic approaches, which simply require drug administration, using ACR2 requires extra optical equipment and surgery. However, since ACR2 is manipulated by light, researchers can inhibit neuronal activity for any duration with an accuracy of milliseconds, which cannot be achieved by chemogenetic approaches. Regarding subtypes of ACRs, ACR2 exhibits faster closing kinetics than ACR1^25^. This characteristic enables more accurate timing control in the optogenetic manipulation in ACR2 than in ACR1. The *LSL-ACR2* mouse strain will provide a new opportunity for researchers to use the efficient inhibitory optogenetic tool.

We found, in the present study, that unilateral continuous inhibition of the activity of LC-NA neurons was sufficient to induce place preference (Fig. 6c). This finding is consistent with a previous study showing that successive photoactivation of LC-NA neurons with ChR2 decreased place preference in RT-PPT^26^. It is known that stressors can activate LC-NA neurons. On the other hand, the tonic activity of LC-NA neurons can cause anxiety-like and aversion behavior^26^. Therefore, inhibition of the activity of LC-NA neurons might have suppressed such negative emotions and serve as a positive valence.

A possible limitation of the utility of ACR2 is the dependence on Cl^−^ for its function. Some neurons show higher intracellular [Cl^−^] than extracellular [Cl^−^], particularly during development^27–30^. In these cases, the reversal potential of Cl^−^ can be higher than the resting membrane potential, and ACR2 activation can induce depolarization rather than hyperpolarization. Therefore, one should take into account the reversal potential of Cl^−^ of the targeted neurons for optogenetic manipulation using ACR2. Further, one might utilize this characteristic of ACR2 to examine whether the reversal potential is higher than the resting membrane potential of recorded neurons by loose cell-attached recording—when the reversal potential is higher than the membrane potential, light illumination results in outflow of Cl^−^ and the extracellular potential can be decreased, and vice versa.

It should also be noted that ACR2 used in this study is not the soma-targeted version of ACR2 (stGtACR2) reported by Mahn et al.^18^. As such, brief illumination of an axon terminal might cause a transient excitation because of the higher axonal reversal potential of Cl^−^^18, 31^. We indeed observed axonal signals in the hippocampal dentate gyrus of the *NAT-ACR2* mouse brain (Supplementary Fig. S3), which is a region receiving projection from LC-NA neurons^32, 33^. Therefore, the most appropriate way to utilize the *LSL-ACR2* mouse strain is for long-lasting continuous inhibition of neuronal activity, as reported in this study and performed during the RT-PPT. ACR2 expressed in *LSL-ACR2* mice can inhibit the activity of targeted neurons for longer periods of time with relatively weak light intensity, which would not generate heat and phototoxicity. Thus, we believe that the *LSL-ACR2* mouse strain, by crossing with Cre-driver mice, will be useful for studies aimed at demonstrating “necessity” of specific neuronal activity by investigating the physiological role of targeted neurons in vitro and in vivo.

## Materials and methods

### Animals

In this study, *Rosa26-CAGp-LSL-ACR2-eYFP* (*Gt(ROSA)26Sor*^*tm1(CAG-ACR2/EYFP)Ksak*^, or *LSL-ACR2*) was newly generated using a method similar to that used to generate the *CAG-floxed STOP tdTomato* reporter line (MGI: 6192640) in a previous study^34^. Briefly, we constructed the targeting vector containing a CAG promoter^35^, frt flanked pgk-Neo cassette^36^, STOP cassette consisting of the terminator of the yeast His3 gene and SV40 poly-adenylation sequence^37^, cDNA encoding ACR2 tagged with EYFP at the C-terminus from *pFUGW-hGtACR2-EYFP* (Addgene: Plasmid #67877), woodchuck hepatitis virus posttranscriptional regulatory element^38^, and rabbit b-globin poly-adenylation sequence^39^. Two loxP sites were inserted before the frt-Neo cassette and after the STOP cassette^37^. This vector also exhibits 5’ and 3’ homology arms of 4.7- and 5.2-kb, respectively, which target the Xba1 site of intron 1 at the Rosa26 locus^40^. The targeting vector (DDBJ: LC744045) was linearized and electroporated into the RENKA C57BL/6 embryonic stem cell line^41^. G418-resistant ES clones were screened by Southern blot analysis for homologous recombination at the Rosa26 locus. Targeted ES clones were injected into eight-cell stage CD-1, which were cultured to produce blastocysts and later transferred to pseudopregnant CD-1 females. The resulting male chimeric mice were crossed with female C57BL/6 mice to establish the *LSL-ACR2* line. The *LSL-ACR2* mice used in the present study exhibit the Neo cassette. Previous studies showed that there is no difference in Rosa26 reporter expression with or without removal of the Neo cassette^42, 43^. Therefore, we did not test removal of the Neo cassette in the present study. The *LSL-ACR2* mouse strain was raised in an inbred-manner for 6 to 13 generations after introduction into our animal facility. All progenies of *LSL-ACR2* mice crossed with Cre-driver mice showed consistent expression of ACR2 (n = 20 animals). To express ACR2 in NA neurons, *noradrenaline transporter (NAT)-Cre* (*Tg(Slc6a2-cre)FV319Gsat*) mice^14^ and *LSL-ACR2* mice were crossed to generate *NAT-Cre;LSL-ACR2* (*NAT-ACR2*) mice, which were used for optogenetic experiments (total 14 animals) and immunohistochemistry (3 animals). To express tdTomato in NA neurons, *Ai14* (B6.Cg-*Gt(ROSA)26Sor*^*tm14(CAG-tdTomato)Hze*^/J) mice^42^ and *NAT-Cre* mice were crossed to generate *NAT-Cre;Ai14* (*NAT-tdTomato*) mice, which were used for negative control experiments in vivo (total 6 animals). To express ACR2 in Vglut2-positive glutamatergic, Vgat-positive GABAergic, or DAT-positive dopaminergic neurons, *Vglut2-Cre* (*Slc17a6*^*tm2(cre)Lowl*^)^44^, *Vgat-Cre* (*Slc32a1*^*tm2(cre)Lowl*^)^44^, or *DAT-Cre* (*Slc6a3*^*tm1.1(cre)Bkmn*^)^45^ mice were crossed with *LSL-ACR2* mice to generate *Vglut2-Cre;LSL-ACR2, Vgat-Cre;LSL-ACR2*, or *DAT-Cre;LSL-ACR2* mice, respectively, which were subsequently used to confirm expression (one animal for each strain). To confirm lack of ACR2 expression in Cre-negative animals, *LSL-ACR2* mice (4 animals) and *NAT-Cre* mice (2 animals) were used. Adult mice (aged > 6 weeks) were used. Animals were housed at 23 ± 2 °C with a 12-h light–dark cycle, and feeding and drinking were available ad libitum. All experiments were carried out following the ARRIVE guidelines 2.0^46^ and the Nagoya University Regulations on Animal Care and Use in Research, and were approved by the Institutional Animal Care and Use Committees of the Research Institute of Environmental Medicine, Nagoya University, Japan (approval R210096 and R210729).

### Immunohistochemistry

Animals were perfused with 4% paraformaldehyde (PFA). The brain was removed and fixed in PFA at 4 °C. After 6 h, PFA was replaced with phosphate-buffered saline (PBS) containing 0.05% NaN_3_ (PBS + NaN_3_) and the sample was allowed to sit overnight at 4 °C. On the next day, the brain was embedded in 3% agarose dissolved in PBS + NaN_3_. Agarose was fixed after 30 min, and 40-µm thick brain slices were sectioned using a vibratome (VT1000S; Leica). Brain slices from *NAT-ACR2* mice were collected every 160 µm. Brain slices from *Vglut2-Cre;LSL-ACR2*, *Vgat-Cre;LSL-ACR2*, *DAT-Cre;LSL-ACR2*, *LSL-ACR2*, and *NAT-Cre* mice were collected every 320 µm. Brain slices were washed with PBS-BX (1% bovine serum albumin, 0.25% Triton X-100 in PBS) 3 times every 15 min at room temperature. The slices were then incubated in primary antibodies (rabbit anti-TH (1:1000, AB152, Chemicon) and chicken anti-GFP (1:1000, GFP-1010, Aveslabs)) diluted with PBS-BX overnight at 4 °C. The next day, the slices were washed with PBS-BX 3 times every 15 min at room temperature. The slices were then incubated in secondary antibodies (CF647 conjugated anti-rabbit IgG (1:1000, 20047-1, BTI) and CF488 conjugated anti-chicken IgY (1:1000, 20079-1, BTI)) diluted with PBS-BX at room temperature for 2 h. Next, the slices were washed with PBS-BX 3 times every 10 min at room temperature. Once washed with PBS + NaN_3_, the slices were incubated with DAPI solution (2 µM, 043-18804, Wako) in PBS + NaN_3_ for 1 h at room temperature, followed by washing with PBS + NaN_3_ 3 times every 10–15 min.

### Imaging and image analysis

Images were acquired with a Zeiss LSM 710 inverted confocal laser scanning microscope and a Keyence BZ-X710 fluorescence microscope. To count the number of ACR2-expressing cells, a 10× objective lens was used in the Zeiss LSM 710 with 405-, 488-, and 561-nm argon lasers. To verify positions, a 4× objective lens was used in the Keyence BZ-X710 with DAPI, GFP, and Cy5 filter cubes (Keyence). The ImageJ software^47^ was used for adjustment of brightness and contrast, and quantification of ACR2-expressing cells (Cell Counter plugin). Three brain slices on the right side of 3 different mice were used for cell counting. Cells expressing TH or ACR2, as well as cells expressing both TH and ACR2 simultaneously, were counted.

### Electrophysiology

Animals were anesthetized with isoflurane. After decapitation, the brain was quickly transferred to the frozen cutting solution (containing, in mM, 15 KCl, 3.3 MgCl_2_, 110 K-gluconate, 0.05 EGTA, 5 HEPES, 25 glucose, 26.2 NaHCO_3_ and 0.0015 (±)-3-(2-carboxypiperazin-4-yl)propyl-1-phosphonic acid) with carbogen gas (95% O_2_ and 5% CO_2_). The brain was sliced into 250-µm thick sections using a vibratome (VT1200S, Leica), and transferred into artificial cerebrospinal fluid (aCSF, containing, in mM, 124 NaCl, 3 KCl, 2 MgCl_2_, 2 CaCl_2_, 1.23 NaH_2_PO_4_, 26 NaHCO_3_, 25 glucose) with carbogen gas (95% O_2_ and 5% CO_2_) at 35 °C for at least 1 h, then at room temperature covered with aluminum foil to avoid light exposure. An amplifier (Multiclamp 700B, Molecular Devices) and a digitizer (Axon Digidata 1550B, Molecular Devices) were used for patch clamp recording. The recording chamber was perfused with aCSF saturated with carbogen gas (95% O_2_ and 5% CO_2_) at room temperature. A glass pipette (GC150-10; Harvard Apparatus) was made with a puller (P-1000, Sutter Instrument) and its resistance was between 2.8 and 7 MΩ. The pipette was loaded with K-gluconate-based pipette solution (in mM, 138 K-gluconate, 8 NaCl, 10 HEPES, 0.2 EGTA-Na_3_, 2 Mg-ATP, and 0.5 Na_2_-GTP, pH 7.3 with KOH) for whole-cell recording, or aCSF for loose cell recording. Under an epifluorescent microscope (BX51WI, Olympus), 505 nm LED illumination (74 µW/mm^2^, 100 ms, Niji, Blue Box Optics) was used to visualize native EYFP fluorescence from ACR2-EYFP. We identified LC-NA neurons by a combination of anatomical location, triangular cellular shape, and fluorescence observed in the recording area. In Fig. 2**,** the membrane potential was held at − 60 mV for measuring current deflection. In Fig. 3**,** the membrane potential was held from − 120 to − 40 mV in 20 mV steps with a duration of 700 ms. The voltage deflection was evaluated at a current holding of 0 pA. Clampex 11.0.3 (Molecular Devices) was used to record the data.

### Optogenetic manipulation in the brain slice

Light illumination was delivered through an electronic stimulator (SEN-3301, Nihon Kohden) connected to a light source (470 nm, 3.1 mW/mm^2^ at maximum, Niji, Blue Box Optics). The light intensity was controlled by our original Python programs^48^ with a microcontroller (Arduino Uno R3). In Fig. 2a, we set the delay at 0 s, the interval at 10 s, the duration at 5 s, and the train at three times, and the intensity was automatically adjusted to 1, 2, 5, 10, 20, 50, and 100%. In Fig. 3a–c, we set the delay at 200 ms, the interval at 0 s, the duration at 200 ms, and the train at 1, and the intensity was adjusted to 50, 5, and 2%. In Fig. 4a**,** we set the delay at 0 ms, the interval at 15 s, the duration at 5 s, and the train at 3 times, and the intensity was automatically adjusted to 50, 5, and 2%. In Fig. 2f**,** we set the delay at 0 ms, the interval at 0 s, the duration at 30 s, the train at 1, and the intensity at 100%. In Fig. 5a, we set the delay at 0 ms, the interval at 0 s, the duration at 600 s, the train at 1, and the intensity at 1%.

### Real-time place preference test (RT-PPT)

Animals were implanted with an optic cannula (φ400 µm, 0.39 NA; F0618S04B2P, Kyocera) above the LC unilaterally (tip at 5.6 mm posterior and 0.9 mm lateral to the bregma, and 3.0 mm ventral from the brain surface). One day after the surgery, animals were habituated to experimenters’ hands for 30 s twice per day for at least 1 week. After animals were habituated, a fiber cannula of an animal was connected with an optic fiber cable (φ400 µm, 0.39 NA; M98L01, Thorlabs) with an interconnect (ADAL3, Thorlabs), attached to a light source (M470F3, Thorlabs). The animal was placed in one side of a two-chamber cage (11 cm in width, 13 cm in depth, and 14 cm in height/chamber) with different floor textures (metal mesh and smooth) and wall appearance (black–white stripes and white) and allowed to explore both sides of the chamber for 10 min (baseline ‘OFF’ session). Animal behavior was monitored by a USB camera (ELP-USBFHD05MT-KL36IR, Ailipu Technology). The position of the nose was identified by Deeplabcut-live^49^ with a pre-trained dataset. After the baseline session, the animal was replaced into the home cage, and the duration in each chamber was instantly analyzed. A chamber in which the animal stayed for a longer duration was defined as the preferred chamber. The animal was then placed in the preferred chamber, and allowed to explore the two-chamber cage for 10 min (RT-PPT ‘ON’ session). During the RT-PPT session, continuous light illumination (470 nm, 50–60 µW at the tip of an optic cannula) was delivered while the animal’s nose was in the chamber opposite to the preferred chamber (i.e., non-preferred chamber). Light illumination was controlled via microcontrollers (Arduino Uno R3) and synchronized red LED light, which was not observable from the subject animal, was shown to the camera. After recording, the recorded videos were analyzed with Deeplabcut^50, 51^ offline and the time spent in each chamber was calculated.

### Statistical analysis

Statistical analysis was performed in OriginPro 2020 (OriginLab Corporation). In Fig. 4d, a paired t-test was used. In Figs. 5b, 6e,f, Tukey’s test was used. In Fig. 6c, Welch’s t-test was used. In Fig. 6d, two-way repeated measures ANOVA followed by post-hoc Tukey’s test was used. Quantitative data are shown as the mean ± standard error of the mean.

## Supplementary Information

Supplementary Figures.

## Data Availability

The DNA sequence of the targeting vector is available in the DDBJ database under the accession number LC744045 (http://getentry.ddbj.nig.ac.jp/getentry/na/LC744045/). The other datasets generated during and/or analysed during the current study are available from the corresponding author on reasonable request.
