# Supplementary material for: Cre-dependent ACR2-expressing reporter mouse strain for efficient long-lasting inhibition of neuronal activity
Source: Sci Rep. 2023 Mar 9;13:3966. doi: 10.1038/s41598-023-30907-2 (PMC9998869; doi:10.1038/s41598-023-30907-2)
Supplement: Supplementary file 1 — Supplementary Figures. [file 41598_2023_30907_MOESM1_ESM.docx]

# Supplementary information

# Cre-dependent ACR2-expressing reporter mouse strain for efficient long-lasting inhibition of neuronal activity

Yasutaka Mukai^1,2,†^, Yan Li^1,2,†^, Akiyo Nakamura^1^, Noriaki Fukatsu^1^, Daisuke Iijima^1^, Manabu Abe^3^, Kenji Sakimura^3^, Keiichi Itoi^4^, and Akihiro Yamanaka^1,2,5,6,7,*^

1. Department of Neuroscience II, Research Institute of Environmental Medicine, Nagoya University, Nagoya, 464-8601, Japan
2. Department of Neural Regulation, Nagoya University Graduate School of Medicine, Nagoya, 466-8550, Japan
3. Department of Animal Model Development, Brain Research Institute, Niigata University, Niigata, 951-8585, Japan
4. Department of Nursing, Faculty of Health Sciences, Tohoku Fukushi University, Sendai, 981-8522, Japan
5. Chinese Institute for Brain Research, Beijing, 102206, China
6. National Institute for Physiological Sciences, National Institutes of Natural Sciences, Aichi, 444-8585, Japan
7. Department of Neuropsychiatry, Keio University School of Medicine, Tokyo, 160-8582, Japan
† These authors contributed equally to this work.
* Corresponding author: yamank@cibr.ac.cn

# Supplementary Figures & Legends


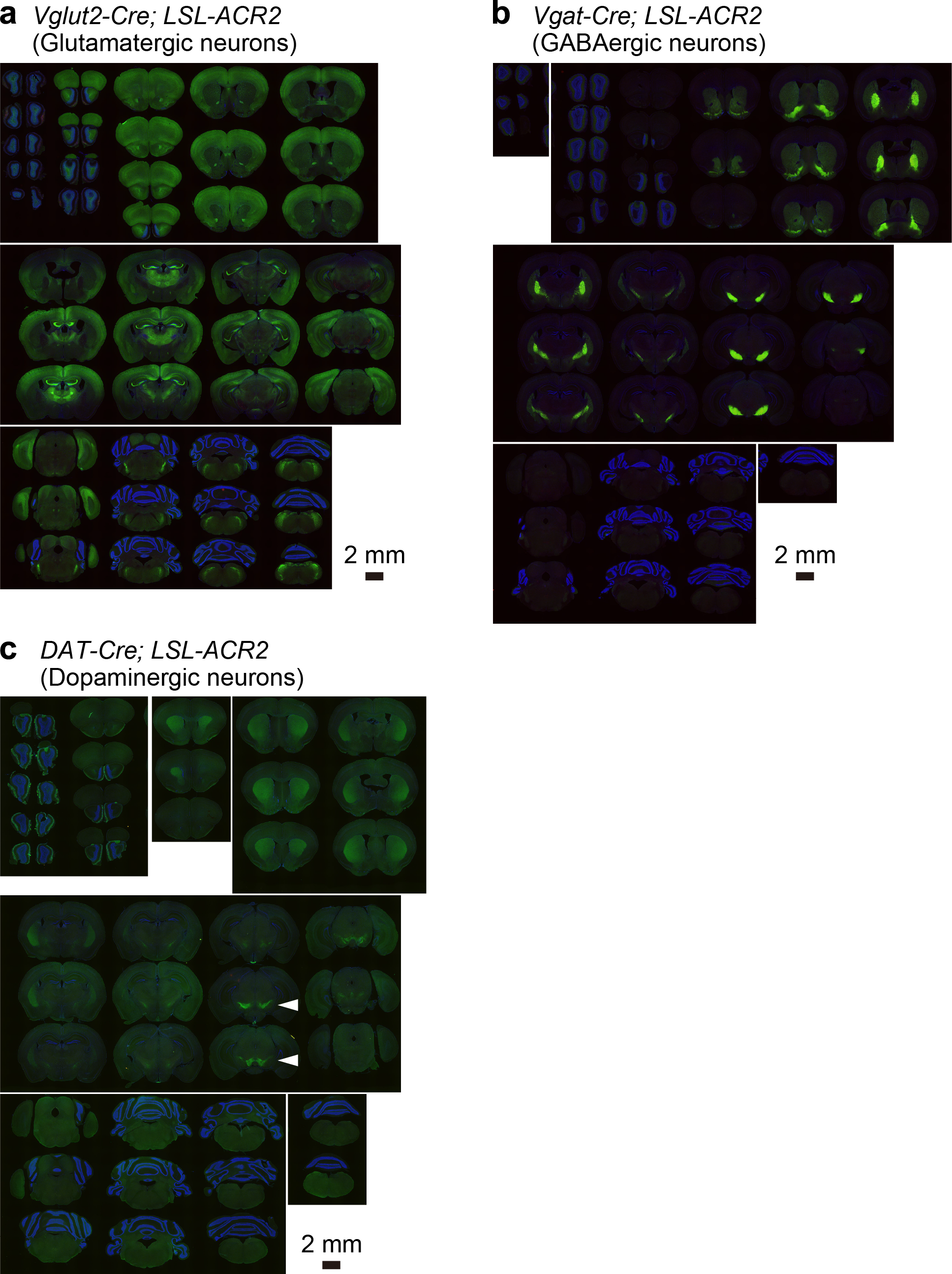


## Supplementary Figure S1. Expression of ACR2-EYFP in siblings of *LSL-ACR2* mice bred with Cre-driver animals

(a–c) Immunostaining images of whole brain slices from *Vglut2-Cre; LSL-ACR2* (a), *Vgat-Cre; LSL-ACR2* (b), and *DAT-Cre; LSL-ACR2* (c) mice. Each animal showed different expression patterns throughout the brain. Blue: DAPI, Green: ACR2, scale bar: 2 mm, arrowheads in (c), putative dopaminergic neurons in the ventral tegmental area.


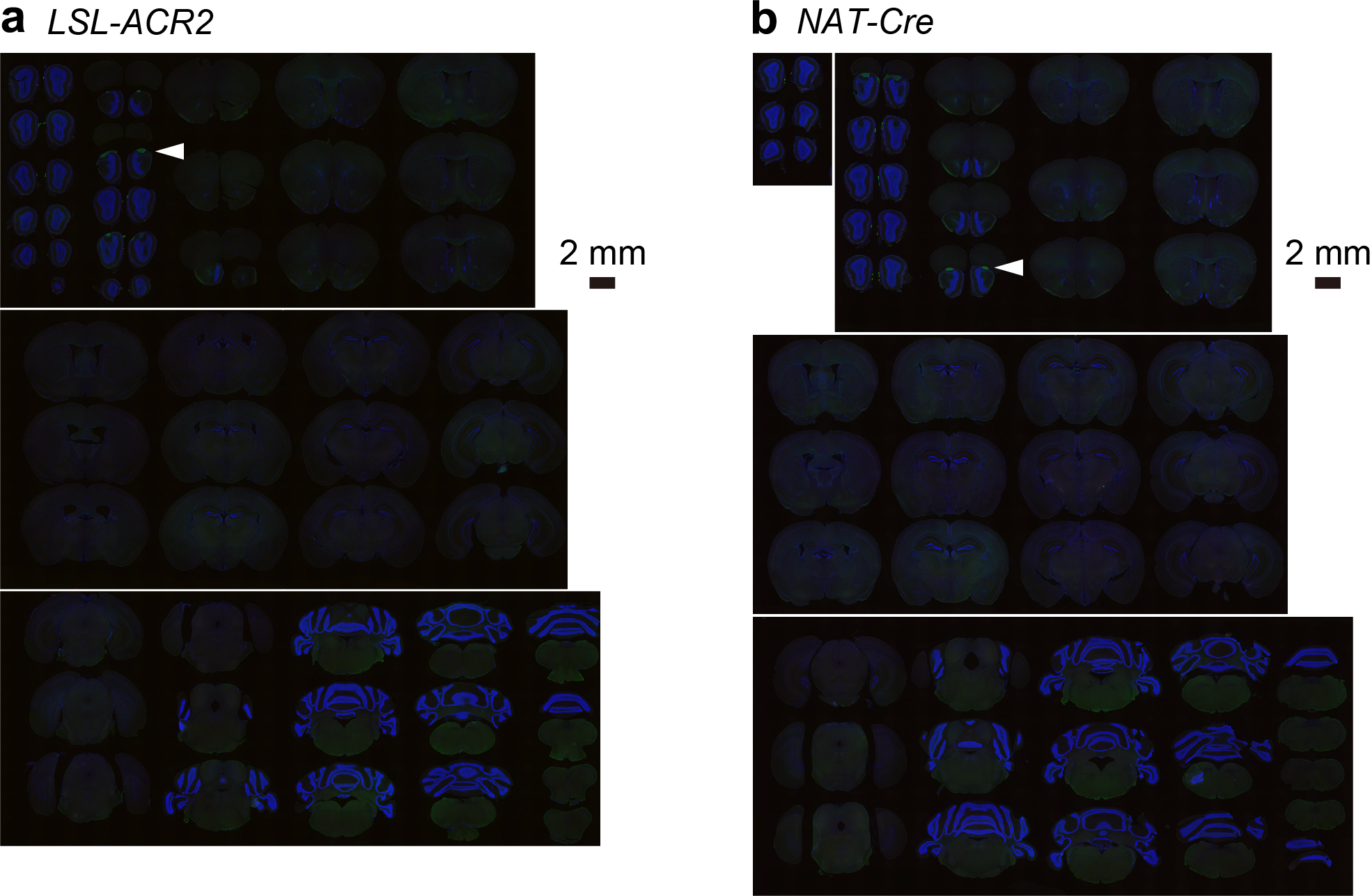


## Supplementary Figure S2. Immunostaining of ACR2-EYFP in negative control animals

(a & b) Representative immunostaining images of whole brain slices from *LSL-ACR2* (a) and *NAT-Cre* (b) mice. Both of animals showed no ACR2-EYFP-derived fluorescence (*LSL-ACR2*, n = 4 animals; *NAT-Cre*, n = 2 animals). Green fluorescence was observed only in the accessory olfactory bulb in both animals (arrowheads), which was not attributed to ACR2-EYFP. Blue: DAPI, Green: GFP-immunoreactivity, scale bar: 2 mm.


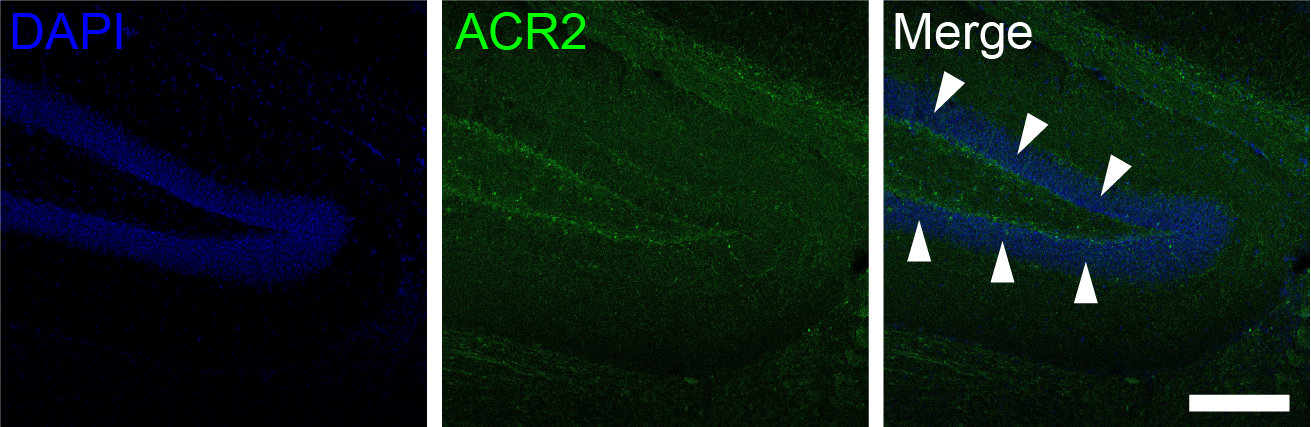


## Supplementary Figure S3. Axonal ACR2-EYFP signal in the hippocampal dentate gyrus of a *NAT-ACR2* mouse

Representative immunostaining images of the hippocampal dentate gyrus (DG) of a *NAT-ACR2* mouse. Axonal ACR2-EYFP signal (arrowheads) was observed in the DG. Blue: DAPI, Green: ACR2, scale bar: 200 µm.
